# Oligodendroglia and Myelin in Neurodegenerative Diseases: More Than Just Bystanders?

**DOI:** 10.1007/s12035-015-9205-3

**Published:** 2015-05-13

**Authors:** Benjamin Ettle, Johannes C. M. Schlachetzki, Jürgen Winkler

**Affiliations:** Department of Molecular Neurology, Friedrich-Alexander University Erlangen-Nürnberg, Schwabachanlage 6, D-91054 Erlangen, Germany

**Keywords:** Oligodendrocytes, Myelin, Oligodendrocyte progenitor cells, Neurodegenerative diseases, Multiple system atrophy, Alpha-synuclein

## Abstract

Oligodendrocytes, the myelinating cells of the central nervous system, mediate rapid action potential conduction and provide trophic support for axonal as well as neuronal maintenance. Their progenitor cell population is widely distributed in the adult brain and represents a permanent cellular reservoir for oligodendrocyte replacement and myelin plasticity. The recognition of oligodendrocytes, their progeny, and myelin as contributing factors for the pathogenesis and the progression of neurodegenerative disease has recently evolved shaping our understanding of these disorders. In the present review, we aim to highlight studies on oligodendrocytes and their progenitors in neurodegenerative diseases. We dissect oligodendroglial biology and illustrate evolutionary aspects in regard to their importance for neuronal functionality and maintenance of neuronal circuitries. After covering recent studies on oligodendroglia in different neurodegenerative diseases mainly in view of their function as myelinating cells, we focus on the alpha-synucleinopathy multiple system atrophy, a prototypical disorder with a well-defined oligodendroglial pathology.

## Introduction

Age-related neurodegenerative diseases are generally characterized by a progressive axonal and neuronal cell loss within circumscribed or multiple regions of the central nervous system (CNS). Extensive efforts have been undertaken to decipher mechanisms underlying the detrimental cascade during axonal and neuronal degeneration and to exploit avenues to modify disease progression. Although various common neuropathological features including disturbances in protein homeostasis, mitochondrial functionality, and axonal transport have been proposed, the precise mechanisms eventually leading to neuronal dysfunction and death still remain elusive [1–5]. Such a comprehensive understanding, however, is the prerequisite for the development of therapeutic approaches attenuating, halting, or even reversing progressive neurodegeneration. As widespread synaptic and axonal loss ultimately leading to neuronal cell death is the common hallmark of neurodegenerative diseases, research addressing disease causing and modifying factors classically focused on autonomous neuronal mechanisms. However, glial cells, namely astroglia, microglia, and oligodendroglia, move more and more into the focus due to their importance for regulating and maintaining neuronal functionality. Moreover and regardless of the distinct disease examined, studying the involvement of glial cells may complement our understanding of these complex diseases. For instance, neuroinflammatory processes mediated mainly by astrocytes and microglia are observed in almost all neurodegenerative diseases [6]. Furthermore, oligodendrocytes (OLGs) and their widely distributed progenitors profoundly influence and control processes shown to be frequently dysregulated in neurodegenerative diseases including ionic homeostasis and nerve impulse conduction [7–11]. Via their densely packed myelin sheaths, OLGs intimately contact and communicate with axons maintaining their integrity as well as regulating axonal and neuronal functionality [12, 13]. Moreover, OLGs are the only mature cellular phenotype within the adult CNS with an existing, widespread, and proliferative progenitor cell population. These oligodendrocyte progenitor cells (OPCs) are not only implicated in OLG replacement and myelin remodeling, but may also serve as a promising source for neuronal replacement [14, 15].

Whereas the literature on oligodendroglial cells in neurodegeneration premillennially comprised only a few studies, the number of reports on this topic has gradually increased over the past few years. Acknowledging the growing evidence of an oligodendroglial contribution to neurodegeneration, the aim of the present review is to highlight recent studies on oligodendroglial cells and their precursors in neurodegenerative disorders. For this purpose, we first reflect on basic aspects of oligodendroglial biology and myelin evolution. Next, we link oligodendroglial and myelin dysfunction to neurodegeneration. Finally, we explicitly dissect the current knowledge on OLGs and their progeny in a distinct neurodegenerative disease by exemplarily considering multiple system atrophy (MSA) as a prototypical entity with a well-known oligodendroglial pathology and even considered to be an oligodendrogliopathy.

## Function and Biology of OLGs and Their Progeny

OLGs represent with 75 % the majority of all glial cells in the adult CNS [16]. The main function of OLGs is the production of myelin, the complex organized and tightly packed enlarged plasma membrane of OLGs wrapped around axons. By insulating axons and clustering sodium channels at myelin-free interspaces—the nodes of Ranvier—myelin enables saltatory action potential conduction. In addition to this well-known function, OLGs have been implicated in various other functions. For instance, OLGs buffer increased extracellular potassium evoked by neuronal excitation, thereby ensuring ionic homeostasis in the CNS [7]. Moreover, OLGs provide metabolic and trophic supply at the axon-myelin unit supporting neurons in maintaining their function [17, 18]. The provision of lactate as energy source via monocarboxylate transporters is essential for axonal survival. By secretion of growth factors, such as glial- and brain-derived neurotrophic factor (GDNF and BDNF), OLGs not only support axonal functionality, but also modulate axonal outgrowth [19–21]. These examples highlight the importance of OLGs and myelin in preserving neuronal circuitries.

Developmentally, OLGs derive from OPCs arising at the ventricular zone and are detected as early as embryonic day 12.5 in the rodent brain. Upon migration during serial waves toward the CNS, OPCs differentiate and give rise to myelinating OLGs [22]. The sequence of differentiation and myelination is a tightly controlled biological process regulated by extrinsic stimuli and a complex transcriptional network. Extrinsic stimuli promoting OPC differentiation and myelination include hormones (e.g., thyroid hormone [23]), growth factors (e.g., BDNF [24]), cytokines (e.g., interleukin-6 [25]), and enzymes (e.g., matrix metalloproteinases [26]). In contrast, several stimuli are described preventing OPC differentiation and myelination, for instance, distinct growth factors (e.g., platelet-derived growth factor (PDGF) [27]) and myelin itself [28]. Transcription factors regulating oligodendroglial differentiation and myelination include amongst others members of the sox family (e.g., sox10 [29]), Olig1 and Olig2 [30], effectors of the Wnt and Notch signaling pathways [31, 32], and the recently identified myelin gene regulatory factor (MYRF) [33].

Although most myelin is produced and laid down during early infancy, OPCs persist in the adult CNS where they are also referred to as NG2 glia (due to their characteristic expression of the neural/glial antigen-2 (NG2)), polydendrocytes, and synantocytes [15, 34]. OPCs are widely distributed in the adult CNS constituting for up to 9 % of all white matter cells and up to 3 % of all gray matter cells in the rodent brain [35]. Lifelong, OPCs keep their potential to proliferate, migrate, and differentiate into mature, myelinating OLGs. Upon demyelination, OPCs undergo this sequential maturation ultimately leading to remyelination [36–38]. Notably, functional differences regarding the differentiation potential between gray and white matter OPC were recently described as white matter-derived OPCs more rapidly differentiated upon transplantation compared to gray matter-derived OPCs [39]. Interestingly, OPCs not only represent an endogenous source for OLG replacement, but also contribute to CNS plasticity [40]. Recent studies demonstrate that OPCs mediate adaptive myelination and motor skill learning in the adult rodent brain [41, 42]. However, this view on OPCs and myelin plasticity was recently challenged in the human brain by analyzing the integration of nuclear bomb test derived (14)C into human myelin [43]. Referring to this study, myelin remodeling conducted by mature OLGs appears to be the main driver of myelin plasticity in the human brain while OPCs make only a minor contribution. Such discrepancy may be related to a significant difference in oligodendroglial biology between humans and rodents.

It is still controversial, whether OLGs are the sole fate of OPCs [44]. Fate-mapping studies in the adult mouse brain demonstrate a few OPC-derived neurons in addition to newborn OLGs [45–47]. Several other reports, however, argue against the neurogenic potential of OPCs [48–50]. The ability of OPCs to obtain neuronal phenotypes might depend on distinct differences between physiological and diseased microenvironments [44]. Nevertheless, OPCs may serve as cellular reservoir and, thus, as a promising therapeutic target not only for OLG replacement, but also for neuronal regeneration as indicated by studies using genetic or pharmaceutical modification to induce the neurogenic potential of OPCs [51, 52]. In addition to their role as progenitors, further regulatory functions have been proposed for OPCs. For instance, it was recently demonstrated that OPCs—synaptically linked to neurons—react activity-dependent to neuronal input with cleavage of the NG2 proteoglycan, thereby modulating the neuronal network [53]. Taken together, OPCs, OLGs, and myelin represent multifunctional constituents essential for the integrity of neuronal circuits. In the following section, we dissect evolutionary and developmental aspects of oligodendroglia and myelin to underline their importance for the extraordinary achievements and the functionality of the human brain.

## Evolutionary and Developmental Aspects of Human Myelin

Undoubtedly, the most important function of OPCs and OLGs is the production of myelin. In fact, myelin represents the most recent crucial step during evolution of the vertebrate CNS appearing first in hinged-jawed placoderms [54]. The rise of myelination is the evolutionary response to the growing size of the vertebrate brain, and thus, it ensures proper neuronal connectivity by facilitating action potential conduction. Myelinated axons require lower currents for signal transduction, and thus, smaller ionic imbalances have to be balanced upon neuronal excitation leading to a major energy economization [55]. Moreover, by accelerating conductivity, myelination is fundamental for the evolution of higher brain functions such as cognition [56]. In addition to myelin, action potential velocity is mainly determined by the axonal diameter. Without the evolution of myelin, axons extraordinary large in diameter would have been required to allow for increasing conductivity. Therefore, spatial optimization is also attributed to myelin [55]. Hence, myelin is an essential factor contributing to the extraordinary accomplishments on neuronal networks in higher organisms such as humans. Considering the fact that neurodegenerative diseases are unique to humans—since not observed in nonhuman primates and other mammals—the development of age-related neurodegenerative diseases may be linked to distinct features of the human brain—such as myelin. Intriguingly, the complexity and upscaling of the human brain are rather reflected by the exceptionalism of its myelin than by increased neuronal and glial cell numbers. Whereas the gray matter volume proportionally evolved in nonhuman primates and humans, the white matter volume especially in the prefrontal cortex is disproportionally larger in humans [57, 58]. In fact, myelination correlates not only with the evolution of advanced brain functions, but also with functional brain development and maturation in each individual. For instance, myelination starts around 25 weeks of gestational age and peaks during early infancy, thus coinciding with the rapid cognitive and emotional development during childhood [59–62]. Moreover, neocortical myelination not only more rapidly progresses in the developing human brain, but also protracts far more into the adulthood in humans compared to nonhuman primates at least partly explaining the extraordinary cognitive, emotional, and social achievements linked to the human brain [63, 64]. Importantly, myelin levels gradually increase until the fifth decade in the human brain before progressively declining in the aging brain as suggested by magnetic resonance imaging (MRI) analyses [65]. As this progressive breakdown of myelin inversely correlates with the increasing risk for the development of neurodegenerative disorders, we next link oligodendroglia/myelin dysfunction to the onset of neurodegeneration.

## Oligodendroglial and Myelin Dysfunction: a Link to Neurodegeneration?

Considering the facts that myelin (i) represents an essential factor for human brain connectivity, (ii) is extraordinarily evolved in humans compared to nonhuman primates and other mammals, and (iii) progressively declines in the aging human brain, oligodendroglial dysfunction significantly contributes to the distinct vulnerability of the human brain for neurodegenerative diseases. Although it is not very likely that myelin dysfunction and oligodendroglial failure are primarily involved in the etiology of neurodegenerative diseases, interindividual variability in OLGs, their progenitors, and/or myelin may define the topographical level of resilience for axonal and neuronal loss. From another point of view, preserved oligodendroglial and myelin functionality may be a crucial prerequisite for the prevention of axonal and neuronal degeneration, e.g., under oxidative or metabolic stress. This causal relationship is supported by several recent studies linking oligodendroglial and myelin dysfunction to axonal and neuronal degeneration.

Strong evidence for an essential contribution of dysmyelination/demyelination to neurodegeneration is derived from studies on multiple sclerosis (MS), one of the best-characterized demyelinating disorders of the CNS [66]. Although the initial MS pathology is tightly linked to autoimmune processes followed by focal demyelination, it is important to note that axonal damage and loss of neuronal integrity represent the structural correlates for the progression of functional deficits in MS patients [67, 68]. Both in human MS brains and the cuprizone-induced demyelinating model for MS, severe axonal degeneration is observed as a consequence of myelin loss [69, 70]. Intriguingly, while no significant axonal damage is observed in remyelinated shadow plaques, severe axonal degeneration is present in active and inactive demyelinated lesions [71]. This indicates that myelin regeneration supports axonal survival. In line, the group of Nave and colleagues demonstrated that the formation of compact myelin is not sufficient for axonal survival. Mice lacking the myelin-specific proteolipid protein 1 assemble physiological myelin sheaths, but develop axonal swellings leading to axonal degeneration. The accumulation of membranous dense bodies and mitochondria within these swellings suggests a profound axonal transport deficiency which may be attributed to the breakdown of the trophic and metabolic support provided by OLGs [72]. Indeed, a more recent study describes pronounced axonal injury upon OLG-specific ablation of monocarboxylate transporters which mediate lactate/pyruvate transport at the myelin-axon junction [18]. These studies in the context of MS highlight the importance of myelin for maintaining axonal integrity and functionality by providing trophic and metabolic support [18, 72, 73].

Furthermore, a notable role was also described for OPCs in MS. Kuhlmann and colleagues observed an accumulation of OPCs in early MS lesions whereas significantly fewer OPC numbers were detected in chronic demyelinated plaques. While Olig2/NogoA double-positive maturating OLGs were present in early lesions, such cells were rarely observed in chronic lesions [74]. Moreover, OLGs which failed to myelinate axons were detected in chronic demyelinated plaques [75]. Considering that axonal damage frequently and predominantly occurs within demyelinated plaques, these findings strongly indicate that a deficiency in both OPC maturation and remyelination contributes to axonal and finally neuronal degeneration in MS [74, 75]. One possible explanation for this self-repair failure is the elevated burden of oxidative and nitrosative stress in MS brains, as these factors were demonstrated to impair OPC maturation [76–78]. In fact, the susceptibility toward oxidative and nitrosative stress is an interesting crosslink between MS and age-related neurodegenerative disease and might act as common denominator in several neurological conditions with white matter alterations [76, 77, 79].

Pioneering studies of Bartzokis and colleagues demonstrated a potential link between myelin breakdown and the onset of neurodegenerative diseases including Alzheimer’s disease (AD) and Huntington’s disease (HD). Using MRI approaches, disturbances of myelin integrity occurring during normal ageing are severely exacerbated in AD and HD [80–82]. Furthermore, increased tissue iron levels—in complex with ferritin which, in turn, is produced in the CNS mainly by oligodendroglia—were measured in AD, HD, and Parkinson’s disease (PD) [9, 11, 82, 83]. Altered tissue iron levels increase the concentration of reactive oxygen intermediates [84]. In turn, this provokes changes in the proteins’ tertiary structure favoring their aggregation propensities and, eventually, neurodegeneration. Therefore, increased tissue iron is considered as risk factor for the development of neurodegenerative diseases further connecting oligodendroglia to neurodegeneration [10, 85]. In addition, white matter abnormalities detected by diffusion tensor imaging (DTI) have been proposed as early event in AD and HD [86–88]. In line, amyloid-beta plaque deposition was recently linked to focal demyelination accompanied by a profound oligodendroglial cell loss, suggesting that demyelination contributes to impaired cortical processing in AD [89]. Studies deciphering oligodendroglial pathology in transgenic mouse models support the notion of an oligodendroglial contribution to AD pathogenesis [90, 91]. In this context, Behrendt and colleagues noted an increased proliferation of OPCs in a mouse model of AD [91]. While this observation favors a cellular response to AD pathology and a potential mechanism for myelin repair, OPC survival, maturation, and myelin sheath formation were negatively influenced by the presence amyloid-beta peptides [92, 93]. Similarly as described in MS, these findings favor a deficiency in remyelination also in AD.

Increased numbers of OPCs indicative for a myelin regeneration failure were also observed in the motor cortex and the spinal cord of amyotrophic lateral sclerosis (ALS) patients, an adult-onset neurodegenerative disease with affection of both the upper and lower motor neuron [94]. Additionally, mice carrying mutations in the SOD1 gene linked to ALS display reactive OPC proliferation upon extensive OLG loss and demyelination even prior to the onset of motor symptoms [49, 94]. The increased number of OPCs was associated with an enhanced differentiation. However, newly generated OLGs exhibited alterations like reduced myelin basic protein (MBP) levels and, thus, were not able to form functional myelin. Indeed, fate-mapping approaches demonstrated that newly formed OLGs were dysmorphic and accumulate with disease progression [95]. Intriguingly, selective ablation of the SOD1 mutation in OPCs and OLGs prolonged survival and delayed disease onset [94]. In line, the ubiquitous reduction of monocarboxylate transporters in OLGs of ALS patients and SOD1 mice implies that the metabolic support of neurons by OLGs is impaired in ALS [18]. These findings indicate an early involvement of oligodendroglia in ALS pathology by increasing neuronal vulnerability.

Taken together, these studies highlight the contribution of myelin dysfunction to neurodegeneration but are, by far, not comprehensive. As the group of neurodegenerative disorders is rather complex and heterogeneous, we next focus on MSA as a neurodegenerative disease with well-known oligodendroglial features.

## Oligodendroglial and Myelin Dysfunction in MSA

### Clinic and Epidemiology of MSA

The term MSA was introduced in the late 1960s combining three initially distinct syndromes: striatonigral degeneration, olivopontocerebellar ataxia, and Shy-Drager syndrome [96]. Indeed, considering the broad spectrum of affected systems, MSA patients symptomatically present with a heterogeneous array of autonomic dysfunction, parkinsonism, cerebellar ataxia, and pyramidal features [97]. MSA is a rare neurodegenerative syndrome with a prevalence ranging between 1 and 5 per 100,000. The incidence is highest in the population older than 50 years reaching a maximum of 3 per 100,000/year [98, 99]. Based on a recent multicenter study in Europe, the mean age of onset is 56 years, while the mean survival was estimated to be about 10 years ranging between 2 and 18 [100]. Thus, MSA is a rapidly progressing neurodegenerative disorder, however, with still unknown etiology. Using whole genome sequencing, a recent study conducted in a Japanese cohort of MSA patients identified heterozygous mutations in the COQ2 gene to cause MSA [101]. However, two follow-up studies in China and in the USA did not detect any heterozygous COQ2 mutations [102, 103]. Nevertheless, common and rare variants of the COQ2 gene were associated with an increased risk for the development of MSA [101–103]. Given these controversial results for the role of the COQ2 locus in MSA, larger cohort studies may help to define the genetic component in MSA. Overall, there is an urgent need for identifying genetic and/or environmental factors underlying MSA pathogenesis, in particular, due to the lack of interventions for MSA patients.

### Human Studies on Oligodendroglia and Myelin in MSA

Neurodegenerative changes in MSA involve predominantly the striatonigral or olivopontocerebellar systems leading to the clinical subtypes MSA-P (with predominant parkinsonism) and MSA-C (with predominant cerebellar features), respectively [104, 105]. In MSA-P, atrophy and gray-greenish discoloration of the putamen as well as loss of pigment from the substantia nigra reflect the involvement of the striatonigral pathway. Atrophy of the cerebellum, pons, and inferior olives is observed in MSA-C brains. Lesions, however, are not restricted to these anatomical regions but involve, in particular, the autonomic nuclei within the spinal cord and brain stem reflecting the broad spectrum of autonomic dysfunction observed in MSA [106]. The neurodegenerative changes are accompanied by white matter abnormalities in the aforementioned anatomical regions. Reduced myelin staining and MBP protein levels were detected [107, 108]. Furthermore, patches of degraded myelin were reported [109]. It is noteworthy to point out that white matter changes are readily detected in MSA patients during lifetime by employing DTI [110, 111]. Specifically, white matter abnormalities were detected in the putamen and middle cerebellar peduncles [112–115].

In 1989, Papp and Lantos described the accumulation of insoluble glial cytoplasmic inclusions (GCIs) as the pathological hallmark of MSA. GCIs were primarily observed within mature OLGs and were thus suggested to be causal for the widespread myelin loss associated with both axonal and neuronal degeneration in MSA [108, 116]. Notably, the vast myelin loss observed in MSA patients is not accompanied by a severe loss in numbers of mature OLGs [117]. Alterations in the glial compartment within the white matter, however, are not restricted to oligodendroglial cells but include astrocytes and microglia. For instance, the extent of reactive astrocytosis parallels the degree of neurodegeneration in the striatonigral and olivopontocerebellar projections [104, 118, 119]. Furthermore, activated microglia phagocytosing myelin are detected within the white matter tracts and are closely associated with the distribution of GCIs [120]. Neuronal cytoplasmic inclusions (NCIs) and neuronal nuclear inclusions (NNIs) are also present in MSA, however, less frequently as GCIs [121]. After revealing GCIs in oligodendroglial cells as defining neuropathological feature of MSA, Papp and Lantos linked the regional GCI pattern to neurological deficits in MSA patients, suggesting that GCI formation in OLGs is rather early and causally involved in MSA pathogenesis [122, 123]. Further support for the pivotal role of GCIs in the pathogenesis of MSA is derived from the correlation of the anatomical distribution between GCIs and neurodegeneration [104, 124, 125]. Moreover, GCI load increases with disease progression [104, 122, 125].

Three almost simultaneously published studies 10 years after the initial description of GCIs revealed alpha-synuclein as the main proteinaceous constituent of GCIs [126–128]. MSA shares the aggregation of alpha-synuclein as a major pathological hallmark with PD and dementia with Lewy bodies (DLB) leading to the classification of these diseases as synucleinopathies [129]. Several additional proteinaceous components of GCIs were defined (for an overview, see Table 1); alpha-synuclein oligomers and fibrils, however, form the central core of GCIs [152]. The group of Jensen and colleagues described disturbances in the interaction between p25-alpha and one of the major myelin proteins, MBP, in the brains of MSA patients as an early event during GCI formation [109]. P25-alpha, also known as tubulin polymerization-promoting protein due to its microtubule-binding activity, is an OLG-specific phosphoprotein. Although its exact function in the myelin sheath is still unknown, p25-alpha expression begins along with MBP and is thus considered as a marker for myelinating OLGs. An ubiquitous cytoplasmic relocalization of p25-alpha which usually strongly interacts with MBP in the myelin sheath was detected. Concomitantly, MBP protein levels severely decreased, indicating that p25-alpha redistribution induces demyelination. In addition, p25-alpha was shown to induce alpha-synuclein aggregation and GCI formation [109, 153, 154].

**Table 1:** Overview of proteins and their major function identified as components of glial cytoplasmic inclusions in multiple system atrophy

Protein	Main function/cellular process	Reference
Alpha-synuclein	Presynaptic vesicle release	[126]
Cyclin-dependent kinase 5 (CDK5)	Cell cycle regulation	[130]
Mitogen-activated protein kinase (MAPK)	Signal transduction	[130]
Midkine	Neurotrophic factor	[131]
Rab5	Endocytosis regulation	[132]
Rabaptin-5	Endocytosis regulation	[132]
P39	CDK5 activator	[133]
Elk1	Transcription factor	[134]
Tau	Microtubule associated protein	[135]
14-3-3 proteins	Signal transduction	[136]
Clusterin/apolipoprotein J	Several functions incl. apoptosis	[137]
Synphilin-1	Alpha-synuclein interacting protein (SNCAIP)	[138]
Dorfin	Protein degradation	[139]
Small ubiquitin-like modifier (SUMO-1)	Protein degradation	[140]
Alpha B-crystallin	Protein folding	[141]
Negative regulator of ubiquitin-like proteins 1 (NUB-1)	Negative regulation of NEDD8	[142]
Parkin co-regulated gene (PACRG)	Regulation of cell death	[143]
P25-alpha	Tubulin polymerization	[109]
DARPP32	Regulation of signal transduction	[144]
HtrA2/Omi	Apoptosis	[145]
Protein disulfide isomerase (PDI)	Protein folding	[146]
Metallothionein-III	Metal binding	[147]
Gamma-tubulin	Microtubule nucleation	[148]
Histone deacetylase 6 (HDAC6)	Tubulin deacetylation	
20S proteasome subunits	Protein degradation	
Heat shock protein 70 (Hsp70)	Protein folding	
Heat shock protein 90 (Hsp90)	Protein folding	
62-kDa protein/sequestosome 1 (p62/SQSTM1)	Autophagy	
NBR1	Autophagy	[149]
F-box only protein 7 (FBXO7)	Ubiquitination	[150]
X-linked inhibitor of apoptosis protein (XIAP)	Regulation of apoptosis	[151]

Additional studies investigating OLG and myelin functionality support the notion of an early oligodendroglial and myelin involvement in MSA. Alterations in the lipid composition restricted to affected regions in MSA brains were recently reported [155]. In line, increased expression of the ATP-binding cassette transporter 8 (ABCA8) involved in lipid transportation in OLGs of MSA patients favors a pivotal oligodendroglial dysfunction in MSA as overexpression of ABCA8 elevates alpha-synuclein and p25-alpha expression [156]. Moreover, GCI-negative OLGs already display increased activity of the endoplasmatic reticulum-associated unfolded protein response [157]. The demonstration of altered oligodendroglial nuclei in absence of GCIs further indicates a general oligodendroglial dysfunction preceding alpha-synuclein aggregation and GCI formation. However, most research focused on the pathogenic role of alpha-synuclein in OLGs during MSA progression [158].

Biochemical and histological studies characterized modifications and solubility of alpha-synuclein demonstrating its abundant alterations in the brains of MSA patients [159–163]. For instance, the accumulation of nitrated alpha-synuclein is observed in MSA, PD, and DLB, and thus, oxidative modifications of alpha-synuclein appear to be common in synucleinopathies [163]. Several groups aimed to decipher the origin of abnormal alpha-synuclein accumulation in OLGs of MSA patients. It is still a matter of debate whether alpha-synuclein is pathologically overexpressed or taken up by OLGs in MSA. Whereas some studies imply the absence of elevated alpha-expression expression, various other reports argue for an elevated alpha-synuclein expression in OLGs of MSA patients [164–168]. It is important to note that mutations or multiplications of alpha-synuclein gene are not detected in MSA patients [169–171]. Polymorphisms within the alpha-synuclein locus, however, are commonly observed in MSA patients possibly explaining altered expression of alpha-synuclein in OLGs of MSA patients [172, 173]. This controversy is supported by studies in animal and cell culture models providing explanations for both the exogenous and the endogenous origin of alpha-synuclein within OLGs of MSA patients [174–181]. Considering these studies, it is reasonable to hypothesize that both autonomously expressed and taken up alpha-synucleins contribute to the cellular phenotype observed in MSA brains.

Although representing a potential cellular source for OLG replacement, studies on OPCs in MSA patients are rare. Two studies recently reported increased numbers of OPCs in MSA patients [182, 183]. May and colleagues additionally demonstrated the presence of alpha-synuclein accumulation within a small number of striatal OPCs [183]. Thus, alpha-synuclein accumulation within precursor cells may disrupt OLG replacement and myelin regeneration contributing to the profound myelin loss observed in MSA. Summarizing the spectrum of imaging and postmortem studies conducted in MSA patients, oligodendroglial and myelin dysfunction is a general feature occurring early during pathogenesis or even being causal for MSA.

### Preclinical Studies on Oligodendroglia and Myelin in MSA

Because aggregated alpha-synuclein is the major constituent of GCIs, generation of preclinical in vivo and in vitro MSA models targets alpha-synuclein expression. Until now, three transgenic mouse lines have been generated, in which alpha-synuclein overexpression in mature OLGs is achieved by driving expression under the control of different myelin gene promoters, namely the 2′,3′-cyclic-nucleotide-phosphodiesterase (CNP) [184], the proteolipid protein (PLP) [185], and the MBP promoter [186]. Independent of the promoter used for oligodendroglial alpha-synuclein expression, typical pathological hallmarks observed in MSA patients including motor impairment and profound myelin loss are recapitulated in all three models [184–186]. Dopaminergic cell loss in the substantia nigra is observed in PLP and MBP mice, while MBP-driven alpha-synuclein expression additionally leads to a reduction of striatal neurons and tyrosine-hydroxylase-positive fibers [185–187]. In contrast to motor dysfunction, nonmotor symptoms including impaired olfaction as well as cardiovascular and urogenital dysfunction are less mirrored in transgenic mice [188–192]. In the PLP-driven model, however, oligodendroglial lesions and neuronal cell loss were recently described in multiple regions controlling autonomic functions matching the occurrence of cardiovascular dysfunctions in this model [189, 193].

The presence of axonal and neuronal cell loss, albeit being far more moderate than observed in MSA patients, as well as similarities to the neurological phenotype in oligodendroglial alpha-synuclein mice, strengthens the view on a causal role of oligodendroglia in MSA pathogenesis. Demonstrated in the CNP model, oligodendroglial alpha-synucleinopathy also induces neuronal alpha-synuclein aggregation by release of the secretory protein cystatin C and, thus, potentially triggers neurodegeneration in a direct manner [194]. Additional evidences for a pivotal role of alpha-synuclein in MSA etiology are derived from distinct mouse lines expressing different levels of alpha-synuclein under the control of the MBP promoter. With increasing levels of alpha-synuclein overexpression, the degree of myelin loss and striatal fiber integrity aggravates. A high level of alpha-synuclein overexpression even causes premature death of transgenic mice resembling the fast disease progression in MSA patients [186]. The involvement of OPCs in MSA is also recapitulated in the MBP model as increased numbers of newborn OPCs were recently described [183]. In this study, the increased number of OPCs was concomitant with a severe myelin loss. Since the numbers of mature OLGs were not altered, this observation suggests a maturation deficit of OPCs preventing OLG replacement and remyelination.

Several studies additionally triggered oligodendroglial dysfunction in transgenic mice overexpressing alpha-synuclein. In order to analyze the contribution of proteolytic failure to oligodendroglia linked pathology in MSA, Stefanova and colleagues systemically inhibited proteasomal activity in PLP-driven alpha-synuclein transgenic mice. Triggered by increased proteolytic stress, oligodendroglial alpha-synuclein accumulation was enhanced, and myelin dysfunction and neuronal cell death associated with an altered motor phenotype were detected supporting the increased vulnerability of OLGs in MSA [195]. An early involvement of proteolytic failure in OLGs during MSA pathogenesis is further supported by in vitro evidences for altered autophagic and proteasomal functionality in OLGs upon alpha-synuclein expression [196]. In line, by enhancing oxidative modifications of alpha-synuclein using the mitochondrial inhibitor 3-nitropropionic acid (3NP), neuropathology and neurological deficits exacerbated in both the PLP- and the MBP-driven transgenic MSA model implying increased susceptibility of alpha-synuclein-bearing OLGs toward oxidative stress [187, 197]. In cultured OLGs, alpha-synuclein aggregation was promoted under oxidative conditions, while increased intracellular alpha-synuclein per se exerted no toxic effect [198–201]. In line, OLG overexpressing alpha-synuclein only underwent apoptosis when co-expression of p25-alpha triggered alpha-synuclein aggregation [202, 203]. Sole overexpression of alpha-synuclein does not promote apoptosis during differentiation of cultured OPC [174]. Additionally, reduced production of reactive oxygen species using selective inhibition of the myeloperoxidase resulted in a profound attenuation of alpha-synuclein pathology, motor impairment, and neurodegeneration [204]. Interestingly, while 3NP injections in wild-type mice caused neuropathological alterations mimicking MSA pathology, alpha-synuclein knockout mice were resilient toward 3NP-induced pathology [205, 206]. These studies imply that alpha-synuclein accumulation and oxidative stress are concomitantly involved in MSA pathogenesis. The selective vulnerability of oligodendroglial cells toward oxidative stress may be partially explained by their extraordinary metabolic requirements for myelin maintenance [207].

Ubhi and colleagues aimed to decipher the contribution of neurotrophic support provided by OLGs for neurodegeneration in the MBP model for MSA. A specific reduction in oligodendroglial-derived GDNF levels was described in MBP mice, while BDNF and insulin-like growth factor-1 levels were similarly reduced in mice expressing alpha-synuclein under neuronal promoters. Restoration of GDNF levels either upon intracerebroventricular infusion or mediated by fluoxetine administration attenuated neurodegeneration, suggesting that reduced trophic support by OLGs significantly contributes to axonal and, ultimately, neuronal degeneration in MSA [208, 209].

Despite the recognition of distinct pathological aspects linked to alpha-synuclein and OLGs in MSA as well as the generation of preclinical models suitable for testing therapeutic interventions, modeling MSA-like pathology by using myelin gene promoter-driven alpha-synuclein expression bears certain limitations. As the currently used MSA mouse models constitutively express human alpha-synuclein, developmentally expressed alpha-synuclein may profoundly contribute to the pathology observed in transgenic mice limiting the validity of interpretations drawn in such studies. Supporting this view, alpha-synuclein is transiently upregulated during development of cultured OLGs [180]. Moreover, two recent studies highlight the detrimental impact of intracellular alpha-synuclein on OPC maturation [174, 183]. In two independent cell culture models, alpha-synuclein overexpression dramatically impaired the maturation of the OPC-like central glia-4 (CG4) cell line and primary rat-derived OPCs demonstrated by reduced upregulation of MBP protein during maturation. Decreasing the level of intracellular alpha-synuclein, however, restored the maturation potential of primary OPCs indicating a tight link between alpha-synuclein and OPC differentiation [174]. Given this interference of alpha-synuclein with OPC maturation, it would be important to establish transgenic mouse lines conditionally expressing alpha-synuclein in order to better model MSA as an age-related neurodegenerative disorder. In fact, the observation of increased OPC numbers, the presence of alpha-synuclein accumulation within a subset of striatal OPCs in MSA patients, and the interference of alpha-synuclein with OPC maturation suggest that not only mature OLGs are affected in MSA demanding the generation of transgenic models expressing alpha-synuclein controlled by more immature oligodendroglial promoters, e.g., PDGF receptor alpha [174, 182, 183].

### Impact of Oligodendroglial and Myelin Dysfunction for MSA Etiology and Progression

**Fig. 1:**
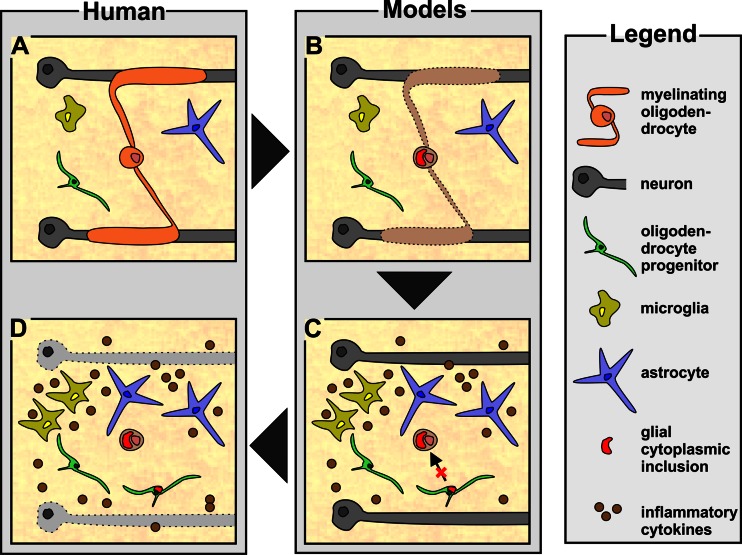
Schematic illustration of the putative pathological sequence underlying MSA pathogenesis. **a** The cellular composition of the healthy human central nervous system is depicted. **b** Dysfunction of myelinating oligodendrocytes with subsequent alpha-synuclein aggregation and glial cytoplasmic inclusion formation is considered as the primary pathological event in MSA. **c** Oligodendrocytic dysfunction and myelin loss induce reactive microgliosis and astrogliosis mediating neuroinflammatory processes. Alpha-synuclein accumulation is also observed in oligodendrocyte progenitors preventing replacement of dysfunctional oligodendrocytes. **d** Ultimately, severe axonal and neuronal degeneration is observed in the central nervous system of end-stage MSA patients

Our current concept on MSA pathogenesis mainly derives from human studies using postmortem brain tissue and preclinical in vitro and in vivo approaches modeling MSA using forced alpha-synuclein expression. The collection of the aforementioned studies (for an overview, see Table 2) shaped our view on MSA assuming a primary oligodendrogliopathy underlying MSA etiology and progression [210]. While conclusions drawn after postmortem analyses in human brains are usually limited to observations on end-stage MSA pathology, experimental models help us to better understand distinct aspects of disease pathogenesis. The pathological sequence illustrated in Fig. 1 summarizes clinical and preclinical data discussed in the present review. Nevertheless, this detrimental cascade described below is still incomplete and may be speculative to some extent.

**Table 2:** Pathological alterations detected in or associated with (A) oligodendrocyte progenitor cells, (B) oligodendrocytes, and (C) myelin in multiple system atrophy and its preclinical models

A. Oligodendrocyte progenitor cells (OPCs)	System/tissue	Reference
Alpha-synuclein accumulation in OPCs	Human: postmortem	[183]
Increased numbers of OPCs	Human: postmortem	[182, 183]
	In vivo: MBP model	[183]
Impaired maturation of alpha-synuclein-expressing OPCs	In vitro: primary and permanent cells	[174, 183]
B. Oligodendrocytes (OLGs)	System/tissue	Reference
GCIs	Human: postmortem	[66, 108, 116]
Alpha-synuclein as major GCI component	Human: postmortem	[126–128, 152]
Modification and insolubility of alpha-synuclein	Human: postmortem	[159–163]
Correlation between GCIs distribution and neurodegeneration	Human: postmortem	[104, 122–125]
Moderate loss of OLGs	Human: postmortem	[117]
	In vivo: CNP and PLP models	[184, 193, 195]
Increased activity of unfolded protein response	Human: postmortem	[157]
Altered morphology of oligodendroglial nuclei	Human: postmortem	[158]
Autophagic and proteasomal dysfunction	In vitro: primary cells	[196]
Increased vulnerability toward proteolytic stress	In vivo: PLP model	[195]
Increased vulnerability toward oxidative stress	In vivo: MBP and PLP models	[192, 197, 204–206]
	In vitro: primary and permanent cells	[198–201]
Reduced neurotrophic support	In vivo: MBP model	[208, 209]
C. Myelin	System/tissue	Reference
Myelin loss	Human: postmortem, diffusion tensor imaging	[107–115]
	In vivo: MBP, and CNP, PLP models	[183, 184, 186, 195]
Altered lipid composition	Human: postmortem	[155]
Altered expression of lipid transport proteins	Human: postmortem	[156]
Protein redistribution	Human: postmortem	[109]

Compared to healthy controls (Fig. 1a), MSA brains are characterized by a profound myelin and neuronal cell loss accompanied by a severe inflammation such as widespread astrogliosis and microgliosis (Fig. 1d) [117, 211]. Additionally, OPCs contribute to MSA pathology as recent studies demonstrate the presence of alpha-synuclein accumulation within OPCs and increased OPC numbers in MSA patients. Experimental evidences suggest that OLGs are dysfunctional early or even initially during MSA pathogenesis (Fig. 1b). This dysfunction may be related to an increased vulnerability of OLGs toward environmental factors (for example, oxidative and proteolytic stress) due to a distinct genetic predisposition. Subsequently, disturbed protein homeostasis within OLGs leads to myelin dysintegrity and the formation of insoluble GCIs consisting mainly of aggregated alpha-synuclein causing demyelination. Neuroinflammation was observed as a consequence of alpha-synuclein overexpression and OLG/myelin dysfunction in MSA mouse models (Fig. 1c) [186, 212, 213]. Triggered by these inflammatory processes and the recently described deficiency in OPC maturation and putatively in remyelination, OLG dysfunction and myelin loss lead to axonal and neuronal degeneration representing the ultimate pathological correlate for the severe clinical phenotype observed in MSA patients.

## Concluding Remarks

In the present review, we highlight the growing understanding on OPCs and OLGs in the context of neurodegenerative diseases. In concert with other glial cells, the contribution of OLGs and their progeny to the widespread axonal and neuronal degeneration is more and more recognized. We aimed to illustrate that oligodendroglial dysfunction is observed in several neurodegenerative diseases, however, being extraordinary in MSA. In this synucleinopathy characterized by its fast and deleterious disease course, the importance of OPCs, OLGs, and myelin for maintaining and supporting neuronal circuitries is evident. Thus, MSA represents a model disease as well as a platform to study the detrimental consequences of oligodendroglial failure for neuronal functionality and survival. Future studies addressing the pathophysiology of OPCs/OLGs and myelin in different neurodegenerative disorders will broaden our understanding of these diseases and potentially open novel avenues for interventional strategies. As OLGs are the only adult cell population with a widespread and proliferative progenitor population, promoting OLG replacement and remyelination may be a promising therapeutic target. In similarity to the extensive efforts already undertaken in the context of MS, enhancing OLG and myelin regeneration in MSA and other neurodegenerative diseases including AD and ALS may support axonal and neuronal maintenance and, thus, attenuate or even halt disease progression.
